# Electrodeless Synthesis of Low Dispersity Au Nanoparticles and Nanoclusters at an Immiscible Micro Water/Ionic Liquid Interface

**DOI:** 10.3390/nano12162748

**Published:** 2022-08-11

**Authors:** Reza Moshrefi, Talia Jane Stockmann

**Affiliations:** Core Science Facility, Chemistry Department, Memorial University of Newfoundland, 45 Artic Ave., St. John’s, NL A1C 5S7, Canada

**Keywords:** Au nanoparticles, nanoclusters, redox active ionic liquid, liquid/liquid electrochemistry, electrosynthesis

## Abstract

Owing to their biocompatibility, optical, and catalytic properties, Au nanoparticles (NPs) have been the subject of much research. Since smaller NPs have enhanced catalytic properties and NP morphology greatly impacts their effectiveness, controlled and reproducible methods of generating Au NPs are still being sought. Herein, Au NPs were electrochemically generated at a water|ionic liquid (w|IL) immiscible micro-interface, 25 µm in diameter, using a redox active IL and compared to results at a water|oil (w|o) one. The liquid|liquid interface is advantageous as it is pristine and highly reproducible, as well as an excellent means of species and charge separation. In this system, KAuCl_4_ dissolved in the aqueous phase reacts under external potential control at the water|P_8888_TB (tetraoctylphosphonium tetrakis(pentafluorophenyl)borate) with trioctyl(ferrocenylhexanoyl)phosphonium tetrakis(pentafluorophenyl)borate (FcIL), an electron donor and redox active IL. FcIL was prepared with a common anion to P_8888_TB, which greatly enhances its solubility in the bulk IL. Simple ion transfer of AuCl_4_^−^ and AuCl_(4−γ)_(OH)_γ_^−^ at the w|P_8888_TB micro-interface were characterized voltammetrically as well as their heterogeneous electron transfer reaction with FcIL. This interfacial reaction generates Au NPs whose size can be thermodynamically controlled by modifying the pH of the aqueous phase. Critically, at low pH, nanoclusters, <1.7 nm in diameter, were generated owing to inhibited thermodynamics in combination with the supramolecular fluidic nature of the IL microenvironment that was observed surrounding the as-prepared NPs.

## 1. Introduction

Au nanoparticles (NPs) have been of continued interest owing to their unique optical and (electro)catalytic properties [1,2] as well as for biomedical applications [2]. Over the past three decades, multiple, relatively straightforward chemical means of generating low dispersity, small (<20 nm) Au NPs have been developed. Many of these have exploited an immiscible liquid|liquid interface as a means of species and charge separation. For example, the Brust–Schiffrin method [3] originally employed the water|toluene interface in conjunction with BH_4_^−^ as a reducing agent and an alkanethiol (RSH) as a capping agent; however, it was later revealed by Uehara et al. that alkanethiols role was far more complicated such as the formation of [Au(I)SR]*_n_* species [4].

**Scheme 1:**
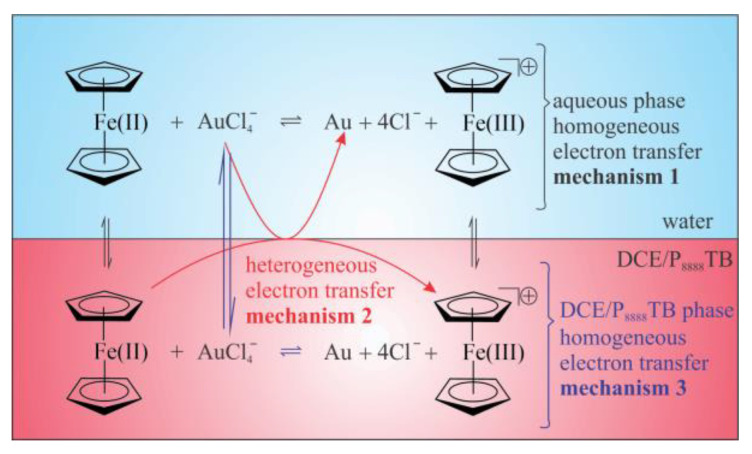
The three electron transfer mechanisms considered between ferrocene (Fc) (or other metallocene derivatives) and AuCl_4_^–^ at a water|1,2-dichloroethane (w|DCE) or w|P_8888_TB (tetraoctylphosphonium tetrakis(pentafluorophenyl)borate) interface.

Moreover, the liquid|liquid interface, or immiscible interface between two electrolyte solutions (ITIES), has become of increasing interest as a platform for the electrodeless synthesis of a variety of materials [5,6,7,8,9,10,11,12,13,14,15,16,17,18]. While at first this relied on spontaneous chemical reactions, increasingly, electrochemical control via an applied external potential is being turned to. This is achieved through immersion of two electrodes, one in either electrolyte phase, so that the Galvani potential difference is localized across the liquid|liquid interface (i.e., *ϕ_w_* − *ϕ_o_* = $\Delta_{o}^{w}\phi$) [2]. Cheng and Schiffrin [19] demonstrated the first electrochemically controlled Au NP generation at an ITIES; whereby, tetraoctylammonium tetrachloroaurate (TOAAuCl_4_) was dissolved in 1,2-dichloroethane (DCE) and was used as both the source of Au as well as an electron acceptor, while potassium hexacyanoferrate(II) (K_4_Fe(CN)_6_) in the aqueous phase served as the electron donor. Thus, the authors were able to exploit the hydrophobicity and hydrophilicity of the electron acceptor and donor, respectively, and limit electron transfer to a heterogeneous process localized across the ITIES. Soon after, Johans et al. [20,21,22,23] began investigating the thermodynamics of nanoparticle generation at an interface with seemingly few nucleation sites. TOAAuCl_4_ is a special case and most contemporary studies have employed a hydrophilic metal salt (e.g., KAuCl_4_ or CuSO_4_), paired with a hydrophobic electron donor (e.g., ferrocene (Fc) [14,15] or decamethylferrocene [16]). In this way, the interfacial or heterogeneous electron transfer pathway can be described as shown in Scheme 1 (mechanism 2). Partitioning of Fc into water (mechanism 1) or AuCl_4_^−^ into oil (mechanism 3) with subsequent homogeneous electron transfer is also depicted.

These previous reports have focused on the water|oil (w|o) interface; however, recently, the water|ionic liquid (w|IL) one has emerged [7,8,9,10,24,25]. Ionic liquids (ILs) are large—on a molecular scale—organic salts with melting points typically below ambient temperature whose molecular architecture can be tuned to obtain a wide variety of physicochemical properties. They are desirable for several reasons including excellent thermal and electronic stability as well as enhanced catalytic properties and as a medium for NP preparation [26]. Indeed, NPs prepared in an IL phase are typically small (<10–20 nm) and have low dispersity; this is hypothesized to be owing to the supramolecular fluidic nature of ILs, which contain nanoscale pockets sandwiched between ion aggregates/contact ion pairs within which NPs can grow [26,27,28,29,30]. Nishi et al. [31] first investigated electrochemically controlled Au NP synthesis at a micro w|IL interface using tri-*p*-tolylamine as the reducing agent dissolved in the IL, trioctylmethylammonium bis(nonafluorobutanesulfonyl)amide ([TOMA^+^][C_4_C_4_N^−^]), and were able to generate nanodendrites. However, in order to elucidate electron transfer between the w and IL phase, Nishi et al. [31] employed an ECSOW (electron conductor separating an oil/water phase) system. In ECSOW, the IL and w phases are not directly in contact; however, a metal wire bridged between the two solutions acts as an electrical connection. Next, they employed a redox active IL (RAIL), (ferrocenylmethyl)dodecyldimethylammonium bis(nonafluorobutanesulfonyl)amide ([FcMDDA]^+^[C_4_C_4_N]^−^), to generate Pd nanofiber arrays at the w|RAIL interface through a spontaneous process (i.e., without external electronic control) [9]. In their proposed mechanism, the RAIL partitions into the water phase and undergoes homogeneous electron transfer with H_2_PdCl_4_(aq).

**Figure 1:**
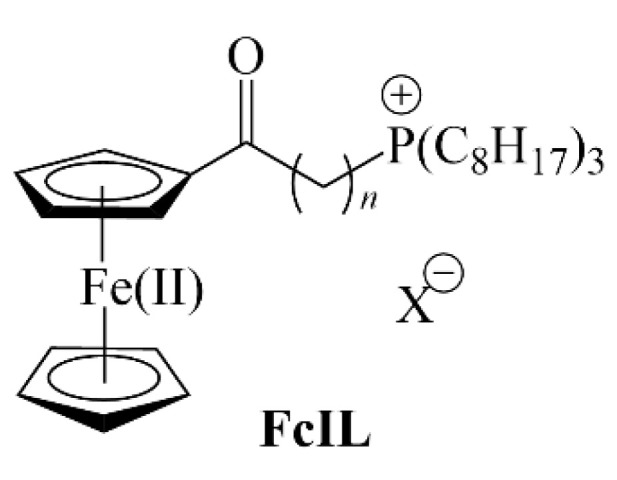
The structure of trioctyl(ferrocenylhexanoyl)phosphonium ionic liquid (FcIL) in which *n* = 5 and X^−^ = B(C_6_F_5_)_4_^−^ (i.e., TB).

Herein, we have investigated electron transfer at a w|IL micro-interface using either of two electron donors, Fc or trioctyl(ferrocenylheptanoyl)phosphonium tetrakis(pentafluorophenyl)borate, FcIL (see Figure 1), in which a Fc moiety is tethered to the phosphonium core by way of an acyl chain. Using either Fc or FcIL dissolved in P_8888_TB (tetraoctylphosphonium tetrakispentafluorophenyl)borate), heterogeneous electron transfer was recorded and resolved at micro-ITIES for the first time without the need to resort to an ECSOW system. Additionally, the influence of aqueous phase pH was interrogated, and low pH elicited thermodynamically inhibited Au NP generation that favored nanocluster formation (i.e., diameters of ~1.7 nm). Additionally, owing to the intermediate hydrophobicity of AuCl_4_^−^ and Fc, by employing the highly hydrophobic electron donor, FcIL, in combination with the P_8888_TB phase, partitioning of either AuCl_4_^−^ or the electron donor was hindered such that mechanisms 1 and 3 in Scheme 1 were not favored.

## 2. Materials and Methods

All chemicals were used as received without purification, while all aqueous solutions were prepared using MilliQ ultrapure water (≥18.2 MΩ cm). Potassium chloride (KCl, >99%), 1-bromooctane (98%), 6-bromohexanoic acid (97%), trioctylphosphine (>97%), tetramethylammonium chloride (TMACl, ≥98%), ferrocene (Fc, >98%), and 1,2-dichloroethane (DCE, ≥99.0%) were sourced from Sigma-Aldrich. Lithium tetrakis (pentafluorophenyl)borate etherate (LiTB, >99%) was purchased from Boulder Scientific. The Fc modified ionic liquid, FcIL (see Figure 1) and P_8888_TB (tetraoctylphosphonium tetrakis(pentafluorophenyl)borate) were prepared as described previously by Weaver et al. [32] and Stockmann et al. [33], respectively.

**Scheme 2:**
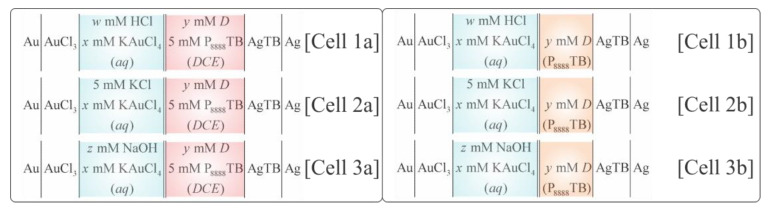
The electrolytic cells employed in which the pH of Cells 1a/b and 3a/b were adjusted using *w* and *z* mM of HCl and NaOH to be pH 2 and 8.5, respectively, as confirmed using a pH meter (sympHony, model#B10P), while *x* and *y* mM of KAuCl_4_ and an electron donor, *D*, were added to the aqueous and DCE or P_8888_TB (tetraoctylphosphonium tetrakis(pentafluorophenyl)borate) ionic liquid phases, respectively. *D* was either Fc or FcIL. The double bar indicates the polarizable potential interface maintained at the pipette tip which, unless otherwise specified, had a diameter of 25 µm. Cells 1b–3b were measured at ~60 °C using a water circulator (Polystat, Cole-Parmer).

A Heka Electroniks PG-618-USB potentiostat equipped with a head-stage was employed for all electrochemical measurements. All measurements were conducted at 25 µm diameter micro-ITIES held at the tip of a micropipette with a scan rate of 0.020 V s^−1^ in a two-electrode mode unless otherwise indicated. The electrolytic cells employed have been drawn in Scheme 2. A specialized micropipette holder was employed with an integrated Au wire, used as the working electrode (WE), and a syringe to back-fill the pipette and maintain the ITIES at the tip. KAuCl_4_ is a powerful oxidant; thus, Ag, or even Pt electrodes were found to be unsuitable as WEs. A CCD camera (AmScope) equipped with a magnifying lens assembly (Navitar) was used to monitor the ITIES position in situ. A second Ag wire (Goodfellow, Inc., Delson, QC, Canada) was immersed in the DCE/P_8888_TB phase and connected to the counter/quasi-reference electrode port of the head-stage.

Micropipettes (25 µm diameter) as well as inlaid disc Pt and carbon fiber ultramicroelectrodes (UME), 25 and 7 µm in diameter, respectively, were prepared as has been described elsewhere [14,27]. UMEs were employed in a two-electrode mode in conjunction with an Ag wire, which served as the counter/quasi-reference electrode.

Transmission electron microscopy (TEM) imaging was performed using a Tecnai Spirit TEM with samples deposited on 200 mesh Au ultrathin/lacey carbon grids.

## 3. Results and Discussion

**Figure 2:**
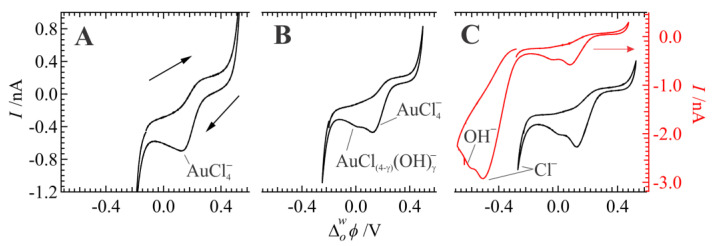
Cyclic voltammograms (CVs) measured using Cells 1a–3a (**A**–**C**) with no electron donor added to the DCE phase. 1 mM of KAuCl_4_ was dissolved in the aqueous phase with the pH = 2, 5.5–6, and 8.5 for the top, middle, and bottom panels, respectively. A 25 µm diameter ITIES was used with a scan rate of 0.020 V s^–1^. The red arrow indicates the axes plotted against, black arrows indicate scan direction, and the peak currents have been labelled with the associated simple ion transfer process taking place.

Figure 2 depicts the cyclic voltammograms (CVs) obtained at a 25 µm diameter interface using 1 mM of KAuCl_4_(aq) in Cells 1a–3a (A–C), or pH 2, 5.5–6, and 8.5, respectively, at the w|DCE interface with no electron donor (*D*) added to the DCE phase (i.e., *y* = 0), with at scan rate of 0.020 V s^−1^. The polarizable potential window (PPW) was limited at positive and negative potentials by the transfer of the supporting electrolyte ions (i.e., K^+^/Na^+^ and Cl^−^/OH^−^), which is seen by the exponential increase or decrease in the current at approximately +0.5 and −0.5 V, respectively [34,35,36]. The use of the micropipette holder makes the system resistant to electrophoretic movement of the micro-ITIES and allows one to scan beyond the usual PPW [2,15,34,36]. In this case, it allows for the observation of Cl^−^ and OH^−^ simple ion transfer processes (see Figure 2C). Simple Cl^−^ transfer was used to reference the potential to the Galvani scale [37].

The *i–V* response at the w|DCE micro-interface is asymmetric; for example, in Figure 2A, during the scan from roughly 0.4 to −0.3 V, a negative peak-shaped wave can be observed with a peak potential at 0.126 V; while during the reverse scan, back toward positive potentials, a sigmoidal wave was recorded with a half-wave potential $\left(\Delta_{o}^{w}\phi_{1/2}\right)$ at 0.172 V. This signal is due to the simple AuCl_4_^−^ transfer from water to oil (w→o) and back from o→w, respectively. The signal asymmetry is owing to the pipette geometry, which elicits hemispherical diffusion outside the pipette and when undergoing charge transfer from o→w and linear diffusion during ion transfer from w→o inside; these curves agree well with previous reports [38,39]. As the pH increased, a second ion transfer wave appeared with a peak potential at ~0.017 V during the negative scan and a sigmoidal $\Delta_{o}^{w}\phi_{1/2}$ at ~0.020 V during the scan towards positive potentials. AuCl_4_^−^ ligand speciation to AuCl_(4−γ)_(OH)_γ_^−^ with hydroxide replacing chloride on the Au core has been well-characterized electrochemically [40] and spectrophotometrically [41]; therefore, the second wave is likely to be due to a combination of a simple AuCl_(4−γ)_(OH)_γ_^−^ transfer consisting of different values of γ. These results are in good agreement with other articles [4,15].

**Figure 3:**
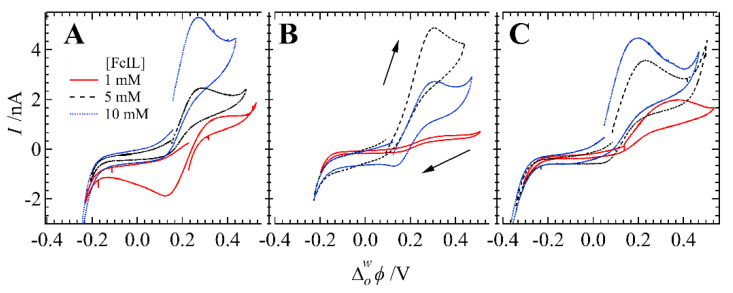
Voltammetric responses for increasing additions of [FcIL], *y* as indicated inset to the DCE phase, with [KAuCl_4_] = 1 mM in Cells 1a–3a or pH 2 (**A**), 5.5–6 (**B**), and 8.5 (**C**). A 0.020 V s^–1^ scan rate was employed with a 25 µm diameter ITIES, while the scan direction is indicated by black arrows.

After addition of 1, 5, and 10 mM of FcIL to the DCE phase in Cells 1a–3a, the negative peak-shaped wave associated with AuCl_4_^−^/AuCl_(4−γ)_(OH)_γ_^−^ disappeared and was replaced by a positive peak signal at ~0.3 V (Figure 3). At [FcIL] = 1 mM and at low pH (red trace in Figure 3A), the AuCl_4_^−^ wave was still present; however, at pH 5.5, a sigmoidal wave was observed in both forward and reverse scans with a $\Delta_{o}^{w}\phi_{1/2}$ of 0.222 V, while at pH 8.5, a positive peak-shaped wave with an onset potential of ~0.15 V has completely replaced the AuCl_4_^−^ transfer signal. At [FcIL] = 5 mM, the onset potential shifted to more negative values by increasing the pH from 2 to 8.5; however, at [FcIL] = 10 mM, the onset potentials were difficult to decern, and this may be owing to a competing homogeneous electron transfer reaction taking place in the DCE phase (see mechanism 3 in Scheme 1).

**Figure 4:**
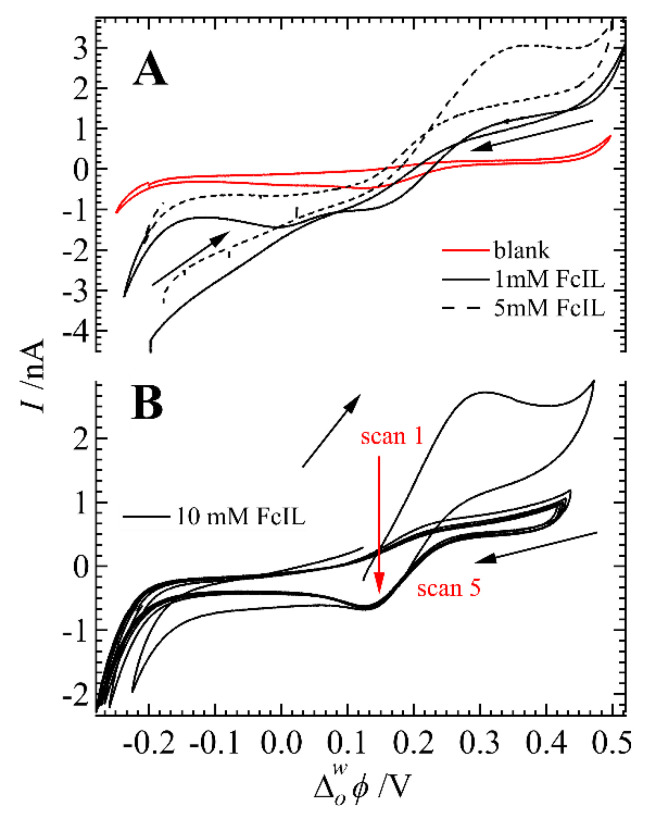
*i–V* measurements using Cell 2a at a 25 µm diameter interface with [FcIL], or *y*, equal to 0, 1, and 5 mM (**A**) as well as [FcIL] = 10 mM (**B**) with multiple scans as indicated inset, performed with a scan rate of 0.020 V s^–1^. Black arrows show scan direction.

Figure 4A depicts the CVs recorded using Cell 2a with 0, 1, and 5 mM of FcIL in DCE and selecting an initial potential near the negative limit of the PPW. With no electron donor added to the DCE phase, the initial current was roughly −0.2 nA; however, with even a modest amount of FcIL added to DCE, a relatively large negative current offset of –3–4 nA was observed. The current offset was still present, even when a 2–5 s potentiostatic pulse was applied just prior to initiating the *i–V* scan. This is likely owing to the spontaneous transfer of AuCl_4_^−^/AuCl_(4−γ)_(OH)_γ_^−^ from w→o, which is caused by the Au salts consumption in a homogeneous, organic phase electron transfer reaction with FcIL (Scheme 1, mechanism 3). This current offset was only observed in the presence of an electron donor in the organic phase. Thus, at high [FcIL], the peak currents and onset potentials in Figure 3 were not reproducible; however, the latter demonstrated a general trend of decreasing toward negative potentials with a concomitant increase in pH.

During the second scan at the w|DCE interface, the positive peak current disappeared (Figure 4B). It is unclear if this is owing to the depletion of the local concentration of FcIL, since the negative peak-shaped wave associated with AuCl_4_^−^ ion transfer seemed to still be present; however, it may be that the intensity of the electron transfer wave only decreased and had taken on a sigmoidal shape.

Owing to the high hydrophobicity of FcIL, it is unlikely that it partitions to the aqueous phase; thus, the positive signal observed in Figure 3 is wholly heterogeneous electron transfer from FcIL in DCE to the AuCl_4_^−^/AuCl_(4−γ)_(OH)_γ_^−^ in the aqueous phase (Scheme 1, mechanism 2). These data agree with our previous results using unmodified Fc at the w|DCE micro-interface [15]. Even as the concentration of the electron donor increases, the electron transfer signal is stable, and large current oscillations were not observed while using FcIL, unlike during our previous work using Fc where large current fluctuations were common [15]. This may indicate that Fc partitioning to the aqueous phase with subsequent homogeneous electron transfer from Fc to AuCl_4_^−^ destabilizes the ITIES. Thus, using a more hydrophobic electron donor decreases the strain on the liquid|liquid interface. Regardless, the Au salt is likely reduced to Au nanoparticles and the oxidized form of FcIL subsequently behaves as a nanoparticle capping agent.

In this way, the following chemical reactions can be written for the heterogeneous electron transfer process:

AuCl_4_^−^(*aq*) + 3FcIL(*org*) → Au(*s*) + 3FcIL^+^(*org*) + 4Cl^−^(*aq*) (1)

AuCl_(4−*γ*)_(OH)*_γ_*^−^(*aq*) + *γ*H^+^(*aq*) + 3FcIL(*org*) → Au(*s*) + 3FcIL^+^(*org*) + (4−*γ*)Cl^−^(*aq*) + *γ*H_2_O(*l*) (2)

where a generalized electron transfer potential at moderate to high pH for the process can be written as [15,42]:

(3) ΔowϕET≈EFcIL+FcILo’,DCE−EAu(III)Auo’,H2O−(0.059 V)3log([H+]γ)

where EAu(III)Auo’,H2O and EFcIL+FcILo’,DCE are the standard redox potentials for AuCl_4_^−^/Au (1.002 V) [43] and FcIL**^+^**/FcIL in the aqueous and DCE phases, respectively. EFcIL+FcILo’,DCE was determined to be 0.91 V using cyclic voltammetry at an inlaid Pt disc UME through a comparison to Fc^+^/Fc (see Figure S1 in the Supplementary Materials, SM). Thus, $\Delta_{o}^{w}\phi_{\mathrm{ET}}^{}$ was calculated to be −0.089, 0.019, and 0.078 V for pH 2, 5.5–6, and 8.5, respectively, assuming *γ* ≈ 1 for pH > 5 and *n* = 3 for the number of *e*^−^ transferred. Thus, as the pH increases, heterogeneous electron transfer becomes more favourable. This agrees well with the general trend of decreasing onset potentials of $\Delta_{o}^{w}\phi_{\mathrm{ET}}^{}$ observed in Figure 3 and with our previous work using Fc [15].

**Figure 5:**
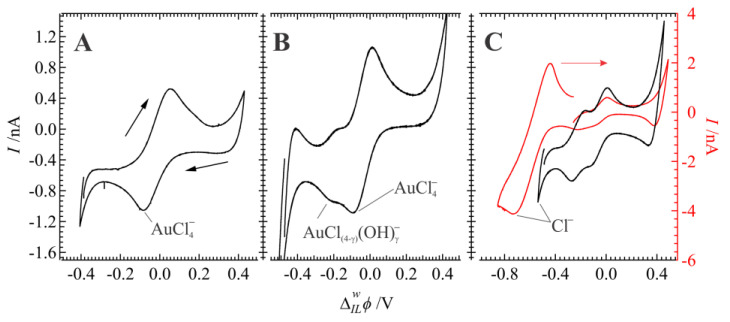
CV responses at a w|P_8888_TB 25 µm diameter interface described by Cells 1b–3b in which the aqueous pH increases from 2 to 5.5–6 and 8.5 (**A**–**C**). Black and red arrows indicate the scan direction and the axes the CV is plotted against, respectively. The cell was maintained at 60 °C using a water circulator (Polystat, Cole-Parmer).

Figure 5A–C show the *i–V* curves obtained if the DCE phase is replaced with P_8888_TB, with no electron donor added, i.e., *y* = 0 mM, and performed at 60 °C using a water heater/circulator. The heater/circulator was connected to a mantle built into the stage and surrounding the vial containing the IL phase. Owing to the high viscosity/low diffusion coefficient in the ionic liquid phase, ion transfer from IL→w elicits a peak-shaped wave rather than a sigmoidal one, i.e., the diffusion regime in the IL phase is linear. These results agree well with previous reports of ion transfer at a w|IL micro-interface [10,33]. The red trace in Figure 5C shows the *i–V* results if the potential is scanned beyond the conventional PPW to reveal simple Cl^−^ transfer with a peak-to-peak (Δ*E*) separation between the forward and reverse scans of ~0.290 V and a half-wave potential, $\Delta_{o}^{w}\phi_{1/2,{\mathrm{Cl}}^{-}}$ ≈ −0.58 V. The latter was calculated as the mid-point between the forward (*E_p,fwd_*) and reverse (*E_p,rev_*) peak potentials for ion transfer (i.e., (*E_p,fwd_* + *E_p,rev_*)/2) while Δ*E* suggests that Cl^−^ is quasi-reversible. Large peak-to-peak separations for redox processes in ILs in the range of 0.1–0.15 V are common [33,44,45] and may be related to the reorganization of the interface on the IL side. It has been shown that the electric double layer (EDL) at the solid|IL [46] and w|IL [47] interfaces is highly organized with alternating anion/cation layers that can extend several times into the IL phase and that have ultraslow relaxation energies. Pushing the PPW far beyond its typical limit likely has a commensurate impact on the EDL relaxation energies exacerbating Δ*E*. As a first approximation, $\Delta_{o}^{w}\phi_{1/2,{\mathrm{Cl}}^{-}}$ was employed to reference the potential to the Galvani scale. Thus, Δ*E* for AuCl_4_^−^ and AuCl_(4−γ)_(OH)_γ_^−^ were measured to be 0.138 and 0.108 V, with $\Delta_{o}^{w}\phi_{1/2,{\mathrm{AuCl}}_{4}^{-}}$ and $\Delta_{o}^{w}\phi_{1/2,{\mathrm{AuCl}}_{\left(4-\gamma \right)}{\left(\mathrm{OH}\right)}_{\gamma }^{-}}$ equal to roughly −0.015 and −0.220 V obtained from the black traces in Figure 5A,C at pH 2 and 8.5, respectively.

**Figure 6:**
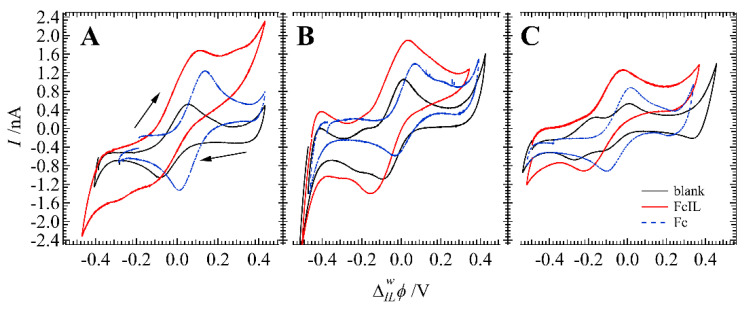
CVs acquired at a 25 µm diameter ITIES between w|P_8888_TB at ~60 °C and 0.020 V s^–1^ using Cells 1b–3b (**A**–**C**) with 1 mM KAuCl_4_(aq) and 100 mM of Fc (blue, dashed trace) or FcIL (red, solid curve) added to the P_8888_TB phase, as shown inset. Black traces depict CVs obtained without an electron donor added to P_8888_TB.

Figure 6 shows the *i–V* curves recorded at the w|P_8888_TB micro-interface (Cells 1b–3b, see Scheme 2) at 60 °C with 100 mM of either Fc or FcIL added to the P_8888_TB phase, as indicated inset, while increasing the pH from 2 to 5.5–6 and 8.5 for panels A–C, respectively. The black traces in Figure 6 are for the system without an electron donor added. At pH 2 (Figure 6A), the peak intensity increased from 1.0 to 1.6 or 2.0 nA with the addition of 100 mM of Fc or FcIL, respectively. At neutral to high pH (Figure 6B,C), the two signals for AuCl_4_^−^ and AuCl_(4−γ)_(OH)_γ_^−^ transfer have been replaced by a single one with peak-shaped waves during the forward and reverse scans with similar increased peak current intensities. Therefore, a similar heterogeneous electron transfer process occurs at the w|P_8888_TB micro-interface and analogous Equations (1) and (2) can similarly be written. No current offset was observed at the w|P_8888_TB interface with the addition of an electron donor, unlike at the w|DCE one (see Figure 4A). The AuCl_4_^−^/AuCl_(4−γ)_(OH)_γ_^−^ transfer potentials at the w|P_8888_TB interface are similar to those observed at the w|DCE one; therefore, these ions have similar affinities towards the P_8888_TB phase as they do toward the DCE one. It is likely that the w|IL EDL organization and high IL viscosity limit the degree of penetration of these ions into the IL phase. Thus, mechanism 3 (see Scheme 1), whereby AuCl_4_^−^/AuCl_(4−γ)_(OH)_γ_^−^ partitions into the P_8888_TB phase and is then consumed by a homogeneous electron transfer with the electron donor, is inhibited. In this way, the w|IL interface can be exploited as a means to restrict electrodeless, electrosynthetic methods to prefer interfacial electron transfer pathways. The half-wave potential for the electron transfer wave $\left(\Delta_{o}^{w}\phi_{1/2,\mathrm{ET}}\right)$ with [Fc] = 100 mM shifted toward more negative potentials of 0.072 V to −0.045 V, with an increase in pH from 2–8.5, while for [FcIL] = 100 mM, $\Delta_{o}^{w}\phi_{1/2,\mathrm{ET}}$ was also found to shift from −0.019 to −0.127 V, with an increase in pH (see Table 1). Δ*E*’s for Fc were between 0.101 and 0.128 V, while for FcIL, the Δ*E* was twice as high, between 0.188 and 0.255 V. These data suggest that electron transfer between the Au salt and FcIL is more thermodynamically favoured versus Fc; however, since FcIL is an IL itself, it likely interacts much more with the IL’s highly organized EDL, which either influences the electron transfer kinetics or disrupts the EDL on the IL side, increasing its relaxation energy. Both possibilities likely contribute to the large observed Δ*E* for electron transfer with FcIL.

**Figure 7:**
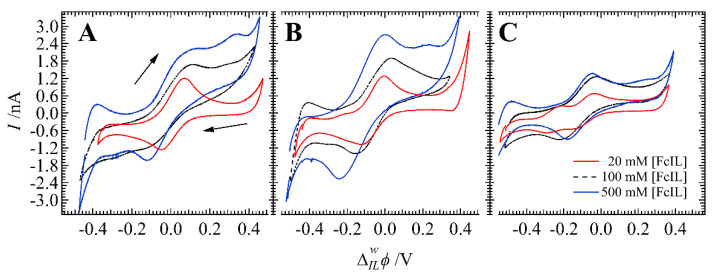
CVs measured using Cells 1b–3b (**A**–**C**) with 1 mM of KAuCl_4_(aq) at a 25 µm diameter interface, 0.020 V s^–1^, and 60 °C, with increasing concentrations of FcIL added to the P_8888_TB phase, as indicated inset.

Figure 7 shows comparison CVs with changing [FcIL] to 20 or 500 mM while altering the aqueous phase pH. At [FcIL] = 20 mM, simple AuCl_4_^−^/AuCl_(4−γ)_(OH)_γ_^−^ is not yet suppressed and likely occurs simultaneously, along with heterogeneous electron transfer. When [FcIL] was increased to 500 mM, two peak shaped waves could be observed on the forward scan towards positive potentials. The second peak at ~0.2–0.3 V may be owing to interfacial coordination of the Fc moiety on FcIL with either H^+^ or K^+^ in the aqueous phase (i.e., a facilitated ion transfer mechanism). Metallocenes have been shown to undergo protonation or lithiation to the cyclopentadienyl ring and likely interact with the water molecules within the hydration shell of K^+^ or other metal cations used as supporting electrolytes [48,49,50]. Generally, as [FcIL] increases, the peak current (*i*_p_) and the totally charge transferred (Q) increase, while $\Delta_{o}^{w}\phi_{1/2,\mathrm{ET}}$ shifts to more negative potentials; the latter is indicative of a more thermodynamically favourable reaction. While the standard redox potentials of FcIL (EFcIL+FcILo’,P8888TB) and Fc (EFc+Fco’,P8888TB) in the P_8888_TB phase are unknown, at low pH, the third term in Equation (3) can be ignored and this can be used as an indirect means for determining EFc+Fco’,P8888TB. Thus, EFc+Fco’,P8888TB was calculated to be 0.99 V vs. SHE. Figure S2 (see SM) depicts the CV obtained at a 7 µm diameter carbon fiber UME immersed in a P_8888_TB solution containing 100 mM of Fc and FcIL; in this way, EFcIL+FcILo’,P8888TB was determined to be ~1.3 V vs. SHE.

**Figure 8:**
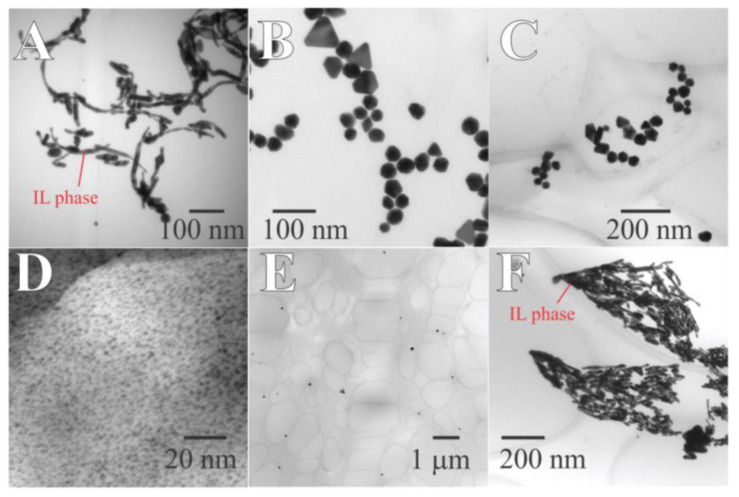
TEM micrographs of samples taken from the aqueous phase after one cyclic voltammetric scan performed at a 25 µm diameter w|DCE (**A**–**C**) or w|IL (**D**–**F**) interface at pH 2, 5.5–6, and 8.5 for the left-hand, center, and right-hand panels, respectively. [KAuCl_4_] = 1 mM throughout; however, [FcIL] = 10 (**A**), 5 (**B**), 20 (**D**,**E**), and 500 mM (**C**,**F**). ‘IL phase’ indicates residual ionic liquid surrounding the NPs.

**Figure 9:**
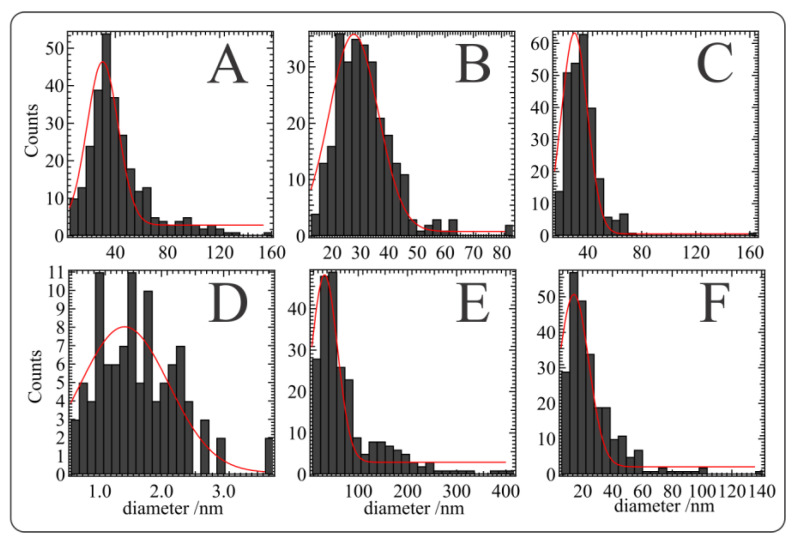
Histograms of nanoparticle diameters measured from TEM micrographs taken after one *i–V* cycle of electrochemical Au NP generation at a w|DCE (**A**–**C**) or w|P_8888_TB (**D**–**F**) at aqueous phase pH of 2, 5.5–6, and 8.5 for the left-hand, middle, and right-hand panels, respectively, with 10 (**A**), 5 (**B**), or 500 mM (**C**) of FcIL in DCE, while 100 mM of FcIL was used in P_8888_TB (**D**–**F**). Red traces are products of Gaussian curve fitting.

At both the w|DCE and w|P_8888_TB micro-interfaces, the aqueous phase was sampled after one *i–V* cycle and a single drop was deposited on a lacey carbon Au TEM grid and imaged. Select TEM micrographs obtained at the w|DCE and w|P_8888_TB interfaces are shown in Figure 8A–F, respectively. Figure 9 shows the nanoparticle size analysis performed for select TEM micrographs using ImageJ software and curve fitting the histograms with a Gaussian function (red traces). The peaks from the Gaussian fittings were taken as the average particle size. At low pH, particles were consistently found with a microenvironment of IL surrounding them (see Figure 8A,D); however, this was also observed occasionally at higher pH (Figure 8F). At the w|DCE interface, at neutral to high pH, nanoparticle sizes were consistent, averaging 27.6 and 30.1 nm in diameter at pH 5.5–6 and 8.5, with [FcIL] = 5 and 500 mM, respectively. With [FcIL] = 100 mM in P_8888_TB at the w|IL interface, average Au nanoparticle sizes were 1.4, 31.1, and 14.0 nm in diameter for pH 2, 5.5–6, and 8.5, as shown in Figure 8D–F, respectively. The concentration of FcIL did not have a large influence on nanoparticle size at either the w|DCE or w|P_8888_TB micro-interface. For example, increasing [FcIL] to 500 mM in the P_8888_TB elicited Au nanoparticles that averaged 20.94 nm in diameter (see Figure S3 in the SM). However, the increase in total charge transferred (Q), as shown in Table 1, indicates that likely more NPs are formed. Our recent results [15] obtained using unmodified Fc at a w|DCE micro-ITIES showed a large dependence on aqueous phase pH with sub-micron particles 400–600 nm in diameter, being generated at high pH, while 20 nm diameter particles were obtained at low pH. This may indicate that mechanism 1 (Scheme 1) is a major component to enhanced NP formation. Thus, the combination of a hydrophobic electron donor and a w|IL micro-ITIES provides control over NP formation and dispersity. Interestingly, when combining a low pH aqueous phase with a w|P_8888_TB micro interface and highly hydrophobic electron donor, one can generate small, low dispersity Au nanoclusters [51]. However, the IL microenvironment seems necessary for the formation of smaller NPs/nanoclusters, which may be owing to its supramolecular fluidic nature [27,30,52].

At low pH, only spherical nanoparticles were seen; however, with increasing pH, lower symmetry nanoparticles were observed with various polyhedrons represented as can be seen in Figure 8B, including octahedral, tetrahedron, and triangular/pentagonal/hexagonal prisms. These shapes agree well with the typical growth pattern of Au nanoparticles [29]. Surfactants have been known to influence the shape of nanoparticle growth [53], and recently, IL computational studies have emerged elucidating the impact of their presence on nanoparticle/nanocluster-IL interactions [54]. Simultaneously, high-resolution TEM has been used to image nanoparticle growth in situ within an IL environment [29]. Based on these images in which an IL microenvironment was associated with smaller nanoparticles/clusters (see Figure 8A) and the fact that the water phase was sampled, it may be concluded that as the particles move into the aqueous phase, they undergo continued growth or Ostwald ripening. Presently, successfully sampling the IL phase for TEM imaging is technically challenging; however, future work will shift to include optical monitoring of NP electrodeless synthesis in situ with an aim to better understand the interfacial dynamics of this process. Nevertheless, these results agree with previous works that indicate that the IL supramolecular fluid plays a critical role in nanoparticle growth [30].

## 4. Conclusions

Herein, an electrodeless synthetic method for generating Au NPs and nanoclusters has been demonstrated exploiting the w|IL micro-interface paired with a second IL with a Fc moiety tethered to the phosphonium core by an acyl chain. The effect of altering the aqueous pH on Au NP growth was investigated by TEM images of droplets extracted from the aqueous phase after a single cyclic voltammetric scan. Importantly, by lowering the pH of the aqueous phase, the thermodynamics of the reaction can be inhibited, favouring Au nanocluster formation (i.e., <1.7 nm in diameter particles). Moreover, the IL supramolecular fluidic microenvironment plays an important role in the size and shape of the particles formed, in agreement with previous works [7,9,29]. Unlike at the w|DCE micro-interface [15], large sub-micron (~500 nm diameter) particles were avoided at high pH. This is likely owing to the inhibition of homogeneous electron transfer, either from the electron donor partitioning into the aqueous phase or by AuCl_4_^−^/AuCl_(4−γ)_(OH)_γ_^−^ transferring to the oil/IL phase (see Scheme 1, mechanisms 1 and 3). Thus, only mechanism 2 (Scheme 1) prevailed.

Increasingly, electrochemical control of the liquid|liquid interface is becoming a favoured method for electrodeless synthesis of novel materials. While this work builds on those fundamental concepts and provides thermodynamic physical insights into NP growth, more importantly, this method affords a novel platform for controlling NP/nanocluster formation and morphology. The latter will be important for widespread manufacture of these materials for catalytic and biomedical applications.

## Figures and Tables

**Table 1:** Data obtained from electron transfer waves depicted in Figure 6 and Figure 7, including the forward (*E_p,fwd_*) and reverse (*E_p,rev_*) peak potentials, the peak-to-peak separation (Δ*E*), and the electron transfer half-wave potential $\left(\Delta_{o}^{w}\phi_{1/2,\mathrm{ET}}\right)$ as well as the peak current (*i*_p_) and total charge transferred (Q) as obtained for the forward wave.

[FcIL]/mM	pH	*E_p,fwd_*/V	*E_p,rev_*/V	Δ*E*/V	$\Delta_{o}^{w}\phi_{1/2,\mathrm{ET}}/V$	*i*_p_/nA	Q/µC
20	2	0.069	−0.051	0.120	0.0086	1.38	38.7
100	2	0.108	−0.147	0.255	−0.0194	2.03	160.2
500	2	0.124	−0.122	0.245	0.00124	2.21	282.2
20	5.5–6	−0.092	−0.125	0.034	−0.109	1.23	123.2
100	5.5–6	0.031	−0.157	0.188	−0.063	1.52	96.0
500	5.5–6	−0.001	−0.243	0.242	−0.122	2.81	265.2
20	8.5	−0.038	−0.160	0.122	−0.099	0.45	41.8
100	8.5	−0.066	−0.266	0.200	−0.127	0.95	160.3
500	8.5	−0.048	−0.182	0.134	−0.115	1.18	166.4
**[Fc]/mM**							
100	2	0.136	0.008	0.128	0.072	1.58	28.3
100	5.5–6	0.068	−0.032	0.101	0.018	1.23	82.4
100	8.5	0.017	−0.107	0.124	−0.045	1.08	21.0

## Data Availability

Data available upon request.

## Supplementary Materials

The following supporting information can be downloaded at: https://www.mdpi.com/article/10.3390/nano12162748/s1, Figure S1: The cyclic voltammetric results at a Pt UME in DCE for the redox reactions with Fc and FcIL; Figure S2: The CVs of the Fc and FcIL redox profiles in P8888TB; Figure S3: Histogram of the Au NP sizes at [FcIL] = 100 mM.
