# Supplementary material for: Electrodeless Synthesis of Low Dispersity Au Nanoparticles and Nanoclusters at an Immiscible Micro Water/Ionic Liquid Interface
Source: Nanomaterials (Basel). 2022 Aug 11;12(16):2748. doi: 10.3390/nano12162748 (PMC9416156; doi:10.3390/nano12162748)
Supplement: Supplementary file 1 [file nanomaterials-12-02748-s001.zip › nanomaterials-1841539-supplementary.pdf]

Electronic Supporting Information for: Electrodeless synthesis of low dispersity Au nanoparticles and nanoclusters at an immiscible micro water/ionic liquid interface

Reza Moshrefi and Talia Jane Stockmann[*]

Memorial University of Newfoundland, Chemistry Department, Core Science Facility, 45 Artic Ave., St. John's, NL Canada A1C 5S7
*Email: tstockmann@mun.ca (T.J.S.)

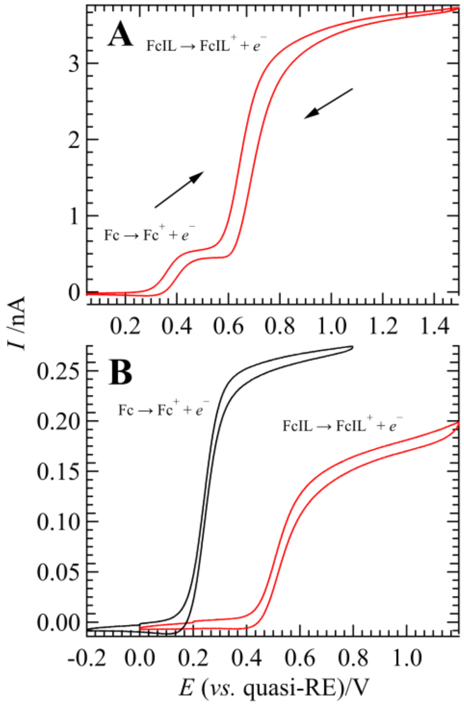

Figure S1: CVs obtained at a 25 µm diameter inlaid disc Pt ultramicroelectrode (UME) immersed in a DCE solution of (**A**) 0.1 mM Fc and 1 mM **1** (see Figure 1 of the main text) or (**B**) individual 50 µM solutions of Fc or **1**. All solutions also contained 5 mM P_{8888}TB as supporting electrolyte, employed a Ag wire as counter/quasi-reference electrode, and were swept at a rate of 50 mV s^{-1}.

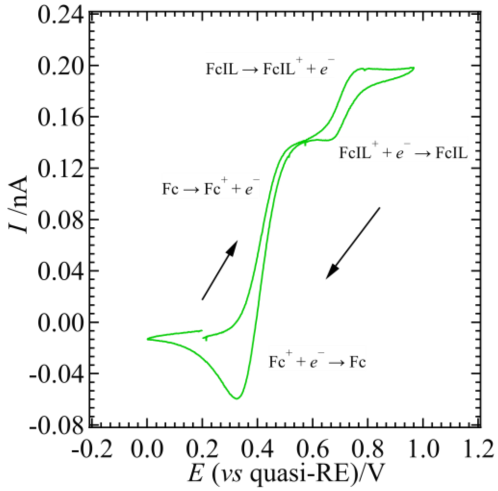

Figure S2: Voltammetric response at a 7 μm diameter inlaid disc carbon fiber UME immersed in P_{8888}TB containing 100 mM of Fc and **1** (FcIL, see Figure 1 of the main text) performed at a rate of 50 mV s^{-1} and ~60°C, while using an Ag wire as a counter/quasi-reference electrode.

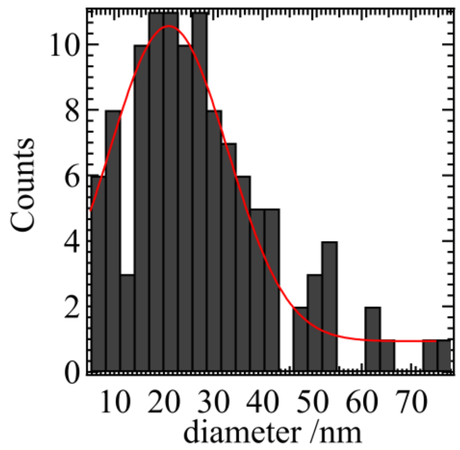

Figure S3: Histogram of Au NP diameters measured from TEM micrographs of aqueous phase sample taken after one *i-V* scan using Cell 3b with 1 mM $KAuCl_4$ and 500 mM of **1** in P_{8888}TB.
